# The Book World of Medicine and Science

**Published:** 1894-10-27

**Authors:** 


### THE BOOK WORLD OF MEDICINE AND SCIENCE.

Prescribing and Treatment in the Diseases of Infants and Children. By Philip E. Muskett. Third edition, revised, enlarged, and re-arranged. (Edinburgh and London: Young J. Pentland.)

This little work, from the pen of one of our Colonial brethren, has just made a re-appearance in the form of a third edition. The care and accuracy with which its pages are written reflects great credit not only on their author but also on the medical school at Sydney to which Dr. Muskett is attached. Very few scientific books—and this is more particularly the case with medical works—which emanate from our Colonies or dependencies deserve a place amongst our permanent text books or works of reference. The work which we hold in our hand has claims on our attention which it is impossible to neglect. It is one which is especially valuable to the young practitioner, whose knowledge of the treatment of children is generally acquired only by experience and is seldom furthered by systematic teaching during his student days. Dr. Muskett's book is arranged in the form of a medical dictionary, that is to say, it is arranged alphabetically, so that for rapid reference it is invaluable; and, further, it is so small and handy that it could be carried in one's coat-tail pocket. As is the case with most of Young J. Pentland's publications, it is a book which is wanted and will sell.

Organic Chemistry. By W. H. Perkin and F. Stanley Kepping. (London and Edinburgh: W. and R. Chambers, L.D. Part I., 3s. 6d.)

Though containing only about 300 pages, this book touches on all the subject matter that is usually dealt with in a course of about sixty lectures. That is to say, it satisfies the requirements of the Science and Art Departments, and is fully sufficient for the organic chemistry examination of the Oxford 1st M.B. and for the chemistry section in the Cambridge Science Papers, where chemistry is taken as an auxiliary subject, and not as the chief subject. The want of some such small text book has long been felt; teachers and pupils have often been compelled to fall back on Remsen's excellent manual; but although this latter is good of its kind, it is a dull and dreary work for the beginner; and its pages are not nearly so attractive in appearance, nor possessed of such valuable and striking finger posts—if one may use the expression—to catch the eye and impress the memory. This, the first part, treats of the fatty compounds, commencing with a clear statement of the methods of separation, purification, and analysis, and a concise account of the determination of the molecular weight. At considerable intervals the letterpress is strewn with paragraphs printed in smaller type; these deal with advanced subjects, which should be omitted by the beginner, and only returned to when the rest of the book has been thoroughly mastered. Although the matter dealt with in these pages is essentially theoretical, it has been treated in as practical a manner as is possible under the circumstances; and to a certain extent the student may in the laboratory follow *pari passu* the stages dealt with in the text. It is a work which will appeal to both teachers and pupils, and will doubtless be the text book of many of our most important teaching departments.

